# *Paraclostridium tenue* Exhibits Antitumor Activity Through Generating Antitumor Metabolites and Modulating Gut Microbiota

**DOI:** 10.3390/cells15090805

**Published:** 2026-04-29

**Authors:** Qianhua Fan, Yao Lu, Huijing Tang, Xiaoying Lin, Ruiting Lan, Shuwei Zhang, Ruoshi Wang, Ruiqing Zhao, Hui Sun, Liyun Liu, Jianguo Xu

**Affiliations:** 1 National Key Laboratory of Intelligent Tracking and Forecasting for Infectious Diseases, National Institute for Communicable Disease Control and Prevention, Chinese Center for Disease Control and Prevention, Beijing 102206, China; lhwfqh@163.com (Q.F.); willongluyao@163.com (Y.L.);; 2 School of Public Health, Nanjing Medical University, Nanjing 211166, China; 3 School of Biotechnology and Biomolecular Sciences, University of New South Wales, Sydney, NSW 2052, Australia; 4 Hebei Key Laboratory of Intractable Pathogens, Shijiazhuang Center for Disease Control and Prevention, Shijiazhuang 050011, China; 5 Research Center for Reverse Microbial Etiology, Workstation of Academician, Shanxi Medical University, Taiyuan 030001, China

**Keywords:** *Paraclostridium tenue*, colorectal cancer, immune factors, metabolites, gut microbiota

## Abstract

**Highlights:**

**What are the main findings?**

- *Paraclostridium tenue* strain Pt517 suppresses the proliferation and promotes the apoptosis of colorectal cancer cells in vitro.
- Oral administration of Pt517 significantly suppresses tumor growth in a CT26 syngeneic mouse model.

**What are the implications of the main findings?**

- The anti-CRC effect of Pt517 is associated with modulation of tumor immunity and remodeling of the gut microbiota.
- The antitumor effects of Pt517 may be mediated via the secretion of long-chain fatty acids, including stearic acid and palmitic acid.

**Abstract:**

Colorectal cancer (CRC) is a digestive tract malignant tumor with a relatively high incidence and mortality rate worldwide. The occurrence and development of CRC are closely associated with disturbances in the gut microbiota. *Paraclostridium tenue* (synonym *Eubacterium tenue*) is generally considered a harmless commensal and can be isolated from fecal samples of healthy adults. However, whether this bacterium is a beneficial organism with an antitumor effect is unknown. This study systematically evaluated the anti-CRC effects of *P. tenue* strain Pt517 on CRC cells in vitro and in the CT26 syngeneic mouse model. Pt517 culture supernatant (Pt517CS) inhibited the proliferation, colony formation, and migration ability of CRC cells; induced cell apoptosis; and altered cell cycle distribution. Daily intragastric administration of Pt517 significantly inhibited tumor growth in mice; increased the expression levels of TNF-α, INF-γ, and CD8 in tumor tissues; and decreased the levels of IL-6, IL-10, and TGF-β. Pt517 intervention significantly modulated the gut microbiota composition with increased relative abundance of *Parabacteroides goldsteinii*, *Lachnospiraceae*, and *Enterorhabdus caecimuris* B7. The long-chain fatty acids (LCFAs), stearic acid and palmitic acid, were increased in the serum of treatment group mice and detected in Pt517CS. Functional verification indicated that stearic acid and palmitic acid directly inhibited the proliferation of CT26 cells in a dose-dependent manner, suggesting that Pt517 might exert its anti-CRC effect by secreting LCFAs. These findings indicate that *P. tenue* Pt517 is a potential new candidate for the microbial treatment of CRC, which warrants further validation for its safety and efficacy before clinical translation.

## 1. Introduction

Colorectal cancer (CRC) is the third most frequently diagnosed malignancy and the second primary cause of cancer-related mortality globally [1]. In recent years, the incidence and mortality of CRC have been on the rise in developing countries [2,3]. In 2020, the estimated new CRC cases were 1.9 million with 930,000 related deaths. It was projected to rise to 3.2 million new cases and 1.6 million deaths by 2040 [4]. Consequently, the prevention and treatment of CRC are gaining growing significance.

Gut microbiota profoundly influences the pathological process of CRC through multiple regulatory mechanisms [5]. Certain pathogenic bacteria, including *Fusobacterium nucleatum* and enterotoxigenic *Bacteroides fragilis*, exert a multifaceted effect on accelerating CRC progression via their virulence factors, intestinal metabolites, and the regulation of immune responses and other relevant mechanisms [6,7]. In contrast, probiotic bacteria such as *Lactobacillus*, *Bifidobacterium*, *Faecalibacterium prausnitzii*, *Akkermansia muciniphila*, and *Eubacterium* exerted regulating immunity and tumor inhibitory effects [8,9,10,11,12,13]. Various studies have found that *Lactococcus lactis* suppressed CRC by reducing the viability of CRC cells, secreting functional protein α-mannosidase, down-regulating cyclin D1, restoring the balance of gut microbiota, and modulating the expression levels of cytokines [14,15,16]. *Bifidobacterium longum* played an inhibitory role in CRC cell proliferation and tumor growth in the AOM/DSS murine model through modulation of oncogenic and tumor suppressor miRNAs, gut microbiota, and immune function [17,18]. *F. prausnitzii* and *A. muciniphila* exerted anti-CRC effects through several mechanisms, including suppressing the proliferation of CRC cells, regulating gut microbiota, modulating the immune system, protecting the intestinal mucosal barrier, and producing short-chain fatty acids (SCFAs) [9,12,13]. Furthermore, *Eubacterium callanderi* inhibited the proliferation of CRC cells and tumor growth of CT26 syngeneic mice by inducing apoptosis and cell cycle arrest, and producing butyrate and 4-aminobutanoic acid, which possess anticancer activity [10]. Our previous study has revealed that *Eubacterium limosum* can inhibit colorectal cancer by modulating tumor microenvironments and generating antitumor metabolites [19].

We initially aimed to study the anti-tumor effect of another *Eubacterium* species, *Eubacterium tenue*. However, *E. tenue* is now shown to phylogenetically belong to the genus *Paraclostridium* [20] and reclassified as *Paraclostridium tenue*. Therefore, this study unexpectedly conducted an assessment of the anti-CRC potential of a member species of *Paraclostridium. P. tenue* strain Pt517 was isolated from the fecal samples of a healthy adult. We found that the culture supernatant of Pt517 exhibited significant anti-proliferative activity against CRC cells. Subsequently, we systematically evaluated the effects of Pt517 on CRC cells in vitro and the CT26 syngeneic mouse model in vivo, and explored its mechanism of action. The results indicated that Pt517 significantly inhibited the proliferation of CRC cell lines, promoted apoptosis, and altered the cell cycle distribution. Meanwhile, in the CT26 syngeneic mouse model, Pt517 affected the expression of immune factors within the tumor, altered the intestinal microbiota composition, and increased the content of metabolites with antitumor effects in the mouse serum, thereby effectively inhibiting the growth of CRC tumors.

## 2. Materials and Methods

### 2.1. Bacterial Strains and Culture

*P. tenue* Pt517 was stored in our laboratory, which was isolated from fecal samples of healthy adults, and species identification was carried out by 16S rRNA gene sequencing and BLAST v2.15.0 search in the NCBI. *Escherichia coli* strain MG1655 (ATCC 700926) was purchased from the American Type Culture Collection (ATCC, Manassas, VA, USA) as a negative control. Pt517 was anaerobically cultured on reinforced clostridial medium (RCM, Hopebio, Qingdao, China) plates at 37 °C for 48 h. MG1655 was aerobically cultured on Luria–Bertani (Thermo Scientific^TM^ Oxoid, Basingstoke, UK) plates at 37 °C for 24 h.

### 2.2. Preparation of Bacterial Culture Supernatant

As done previously [21], after culturing Pt517 and MG1655 in each broth at 37 °C for 48 h, the bacterial culture supernatants were collected by centrifugation at 4000 rpm for 30 min. The supernatants were subsequently filtered through a 0.22 μm filter membrane and stored at −80 °C until further use.

### 2.3. Cell Lines and Cell Culture

Murine colon carcinoma cell line CT26, human colon adenocarcinoma cell lines HT29 and Caco-2, and human normal colon mucosal epithelial cell line NCM460 were purchased from ATCC. Caco-2 was cultured in Dulbecco’s Modified Eagle Medium (DMEM, Gibco, Grand Island, NY, USA) supplemented with 20% (*v*/*v*) heat-inactivated fetal bovine serum (FBS, Thermo Fisher, Waltham, MA, USA), and other three cells were cultured in RPMI-1640 (Gibco, Grand Island, NY, USA) medium supplemented with 10% FBS, and maintained at 37 °C in a 5% CO_2_ incubator, a total of 1–5 × 10^6^ cells were collected from each culture flask per experiment [22,23,24].

### 2.4. Cell Viability Assay

Cell viability was measured by the 3-(4,5-dimethylthiazoly-2-yl)-2,5-diphenyltetrazolium bromide (MTT) assay using a commercially available MTT kit (Solarbio, Beijing, China). The MTT assay reflects cellular metabolic activity by measuring the activity of mitochondrial succinate dehydrogenase, thereby indirectly assessing cell viability and proliferation rather than directly determining cytotoxicity. In this study, the anti-proliferative ability of the samples was indirectly evaluated using the MTT assay. Cells were inoculated in 96-well plates, with 5 × 10^3^ cells in each well, and cultured overnight. The culture supernatants of Pt517 (Pt517CS) and MG1655 (EcCS) were mixed with fetal bovine serum and medium in different proportions to prepare solutions with 10–20% (*v*/*v*) for the cell viability assay. Subsequently, the cells were incubated with these solutions at 37 °C with 5% CO_2_ for 48 h. A microplate reader was used to measure the absorbance at 490 nm, reflecting the quantity of formazan produced from the MTT assay [25]. The proliferation rate of cells in each well was calculated using the following formula: Anti-proliferative ability = (A_blank_ − A_treatment_)/(A_blank_) × 100%, (A = absorbance).

### 2.5. Colony Formation Assay

As previously described, CT26 cells were seeded in 6-well plates at 1000 cells per well overnight, as previously described [22]. The cells were then cultured for 14–21 days after adding 14% Pt517CS, EcCS, or RCM broth (blank control), with the medium replaced every 3–4 days. Following completion of the incubation, cells were rinsed with PBS, fixed in methanol, and stained with 0.5% crystal violet solution. After drying, the plates were photographed, and the number of colonies counted. Only cell clusters with more than 50 cells were defined as a single colony and counted.

### 2.6. Cell Migration Assay/Wound Healing Assay

CT26 cells were seeded in 24-well plates at a density of 1 × 10^5^ cells per well. After 12 h of incubation, when the cells reached 80–90% confluence, a linear scratch was created across the diameter of each well using a 1 mL plastic pipette tip, and the wells were gently washed with PBS to remove detached cells and debris. The cells were then cultured with 14% Pt517CS, EcCS, or RCM broth (blank control) in RPMI-1640 medium for 24 or 48 h, and images of the scratches were captured. The scratch areas were quantified using ImageJ v1.54f software, and the cell migration rate was calculated as: migration rate (%) = (scratch area at 0 h − scratch area at 24 or 48 h)/scratch area at 0 h × 100%.

### 2.7. Apoptosis Assay and Cell Cycle Analysis

CT26 cells were seeded in 6-well plates at a density of 5 × 10^5^ cells per well and cultured overnight. After 12 h, the cells were treated with Pt517CS or RCM broth (blank control) for 48 h. For apoptosis analysis, the proportion of apoptotic cells was assessed using Annexin V-FITC and PI-PE double staining (Becton, Dickinson and Company, Franklin Lakes, NJ, USA) according to the manufacturer’s protocol [10]. Stained cells were analyzed using FlowJo software Version 10, with early apoptotic cells identified as Annexin V^+^/PI^−^ and late apoptotic cells as Annexin V^+^/PI^+^. For cell cycle analysis, cells were first treated with 14% Pt517CS or RCM broth for 48 h and then washed with PBS and fixed in ice-cold 70% ethanol at 4 °C for 24 h [26]. The fixed cells were centrifuged at 1000 rpm for 15 min, washed with PBS, and stained with 1 mg/mL propidium iodide (PI) (Becton, Dickinson and Company, Franklin Lakes, NJ, USA) for 5 min. 20,000 cells were collected by flow cytometer (BD FACSCalibur, Becton, Dickinson and Company, Franklin Lakes, NJ, USA) and cell cycle distribution was analyzed using ModfitLT 5 software (Verity Software House, Topsham, ME, USA).

### 2.8. Animal Experimental Design

Female BALB/c mice, aged 5–6 weeks, were purchased from Beijing Vital River Laboratory Animal Technology Co. (Beijing, China). The mice were housed in a specific pathogen-free facility under controlled conditions (22 °C ± 3 °C, 12 h light/dark cycle). Food and water were supplied ad libitum, with all animals receiving humane care throughout the experiment.

Following 4 days of acclimatization, mice were randomly allocated to three experimental groups, with 6 animals in each group, the negative control group (NC group), the tumor model group (PBS group), and the Pt517 intervention group (Pt517 group). For the PBS and Pt517 groups, the right flanks of the mice were depilated and inoculated subcutaneously with injections of 5 × 10^5^ CT26 cells [27]. For the Pt517 group, mice were orally administered 1 × 10^8^ CFU of Pt517 in 200 μL PBS daily for 15 days, beginning one day post-tumor inoculation. Mice in the NC and PBS groups received 200 μL of PBS by oral gavage daily from the day after tumor inoculation. Tumor dimensions were measured every 48 h using a vernier caliper, with length (L) and width (W) recorded. Tumor volume was calculated as L × W^2^ × 0.5. When tumors reached approximately 2000 mm^3^, mice were euthanized in accordance with animal care guidelines. All mice were subjected to terminal anesthesia via isoflurane overdose (5% *v*/*v*), and dissected for pathological examination. Blood samples were collected via the ocular route and allowed to clot at room temperature for 1–3 h, and then centrifuged at 4 °C and 3000 rpm for 15 min to obtain serum. Serum was stored at −80 °C for subsequent metabolomics analysis. In addition, tumors were isolated, weighed, and collected. The tumor of the mice with moderate tumor volume in the PBS and Pt517 groups was photographed after separation. Mouse cecal contents were collected in sterile tubes and stored at −80 °C for later gut microbiota analysis [9].

### 2.9. Histological Staining

Tumor tissues were fixed in 4% paraformaldehyde for 24 h, embedded in paraffin, sectioned, and deparaffinized [28]. Paraffin sections were subsequently stained with hematoxylin and eosin (H&E) or immunohistochemically stained for Ki-67. Stained sections were imaged using a panoramic slice scanner (3DHISTECH, Budapest, Hungary).

### 2.10. Cytokine Assay

Tumor tissues (0.1 g per mouse) were rinsed with pre-chilled PBS, minced into small pieces, and homogenized on ice for cytokine quantification [27]. The homogenates were centrifuged at 5000 rpm for 5–10 min at 4 °C, and the supernatants were collected. Cytokines, including TNF-α, IFN-γ, IL-6, IL-10, TGF-β, and CD8, were measured using ELISA kits (Dogesce, Beijing, China) following the manufacturer’s instructions.

### 2.11. 16S rRNA Gene Sequence Analysis

DNA was extracted from fecal samples using the QIAamp Fast DNA Stool Mini Kit (Qiagen, Hilden, Germany). 16S RNA gene amplification was performed with barcoded primers. The forward and reverse primers used were 27F (5′-AGRGTTTGATYNTGGCTCAG-3′) and 1492R (5′-TASGGHTACCTTGTTASGACTT-3′), respectively. Sequencing libraries were constructed, and paired-end sequencing was conducted on the Illumina NovaSeq 6000 platform following the standard protocols of Biomarker Technologies Co., Ltd. (Beijing, China) [29]. After quality control, high-quality circular consensus sequences (CCS) were processed for barcode identification. The optimized CCS sequences were clustered at 97% similarity using USEARCH (version 10.0), and operational taxonomic units (OTUs) were assigned for species classification [30]. Species annotation, taxonomic identification, and alpha diversity analysis were performed using the Silva database and RDP Classifier. Species richness and diversity were assessed using alpha diversity metrics, including Chao and Shannon indices. Beta diversity analysis was used to compare microbial community composition and structure among samples. Linear discriminant analysis effect size (LEfSe) analysis was applied to screen for differentially abundant biomarkers (LDA score > 3.0).

### 2.12. Metabolome Detection of Mouse Serum

For metabolite extraction, 400 μL of cold methanol extraction solvent (1:1, *v*/*v*) was added to mouse serum samples to precipitate proteins and extract metabolites, followed by thorough vortexing. Stable-isotope-labeled internal standard stock solutions were spiked into the extraction solvent to enable absolute quantification of metabolites. After several rounds of centrifugation and supernatant collection, the resulting samples were prepared for metabolomic analysis.

LC-MS/MS analysis was performed using a UHPLC system (1290 Infinity LC, Agilent Technologies, CA, USA) coupled to a mass spectrometer (6500+, Sciex, Marlborough, MA, USA) by Shanghai Applied Protein Technology Co., Ltd., Shanghai, China. Chromatographic separation was carried out using a HILIC column (Waters UPLC BEH Amide, 2.1 mm × 100 mm, 1.7 μm) and a C18 column (Waters UPLC BEH C18, 2.1 mm × 100 mm, 1.7 μm) [31].

Analyst software was used to extract the peak of MRM raw data and calculate the content of serum metabolites. After sum-normalization, the processed data were uploaded, then imported into SIMCA-P (version 14.1, Umetrics, Umea, Sweden) for multivariate data analysis, such as orthogonal partial least-squares discriminant analysis (OPLS-DA). The model robustness was assessed with seven-fold cross-validation and response permutation testing. The variable importance in the projection (VIP) value of each variable in the OPLS-DA model was calculated to indicate its contribution to the classification [32]. The *t*-test was performed to compare the levels of serum metabolites between the two groups, and the fold change was calculated. The differential metabolites between groups were screened based on the *p*-values obtained from *t*-tests and the fold change. The statistical results were visualized using volcano plots and heatmaps.

### 2.13. Genome Sequencing, Assembly, and Annotation of Pt517

Genomic DNA of Pt517 was extracted using the Wizard Genomic DNA Purification Kit (Promega, Madison, WI, USA) following the manufacturer’s protocol [33]. Whole-genome sequencing was performed by Shanghai Majorbio Bio-pharm Technology Co., Ltd. (Majorbio, Shanghai, China) using a combination of PacBio Sequel II and Illumina NovaSeq X Plus sequencing platforms. Raw reads were quality-controlled with fastp v0.20.0 [34]. Coding sequences (CDS) on the chromosome and plasmid were predicted using Glimmer v3.02 or Prodigal v2.6.3 [35] and GeneMarkS v4.3 [36], respectively. Predicted CDS were annotated against the KEGG database using BLAST (blastx/blastp 2.2.28+) [37,38]. Based on previous studies [39,40], key genes involved in the biosynthesis of palmitic acid and stearic acid were identified in the Pt517 genome, indicating that Pt517 possesses the capacity to synthesize these two saturated fatty acids. The final assembled genome has been deposited in the NCBI database under accession number PRJNA1259185.

### 2.14. Statistical Analysis

All statistical analyses and statistical graph generation were performed with GraphPad Prism 10. The data are presented as mean ± standard error of the mean (SEM). Two-sample comparisons were carried out via Student’s *t*-test, whereas multiple-group comparisons employed one-way ANOVA. Data were considered statistically significant when *p* < 0.05 (* *p* < 0.05, ** *p* < 0.01, and *** *p* < 0.001).

## 3. Results

### 3.1. Cytotoxic Effects of P. tenue on Colorectal Cancer Cells

**Figure 1:**
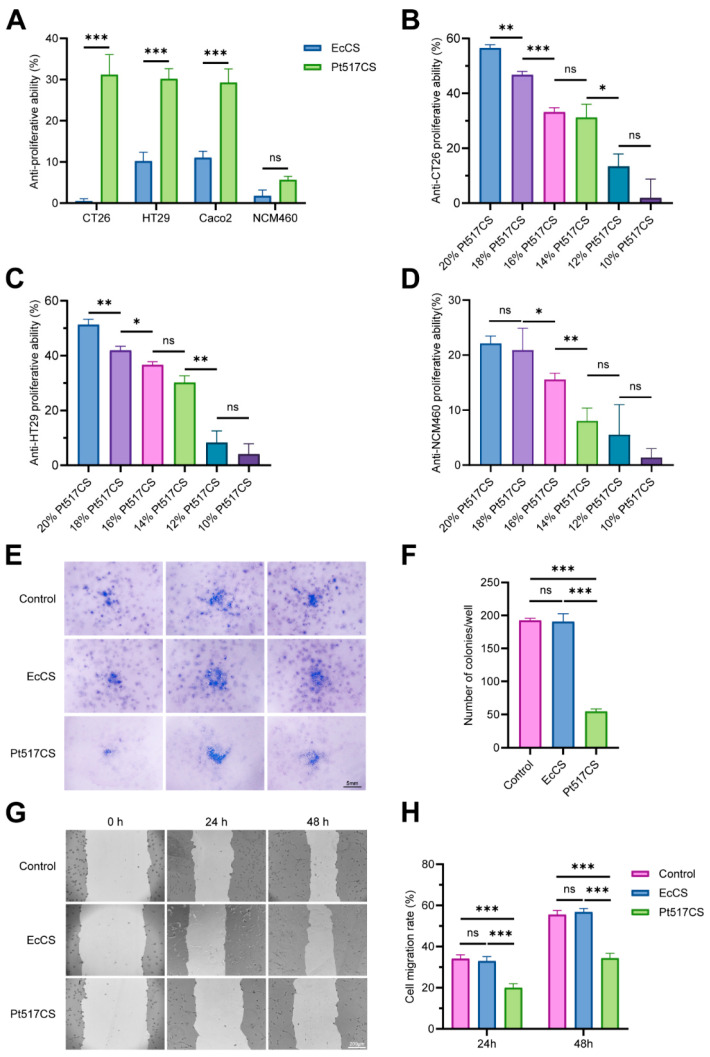
Effects of the culture supernatant of *P. tenue* on the viability of CRC cell lines. (**A**) Effects of Pt517CS on the viability of CT26, HT29, Caco-2, and NCM460 cell lines. (**B**–**D**) Effects of different concentrations of Pt517CS on the viability of CT26 (**B**), HT29 (**C**), and NCM460 (**D**) cell lines. (**E**) Representative images of the cell colonies in different groups. (**F**) Pt517CS significantly inhibited the clone formation of CT26 cell line. (**G**) Representative images of the cell migration in different groups. (**H**) Pt517CS significantly suppressed the migration of CT26 cell line. Data are presented as means ± SEM. *n* = 3 biological replicates. Statistical differences were tested using one-way ANOVA with Tukey’s correction for post hoc testing in (**E**,**F**) and an unpaired two-tailed Student’s *t*-test in (**A**–**C**). ns, not significant, * *p* < 0.05, ** *p* < 0.01, *** *p* < 0.001.

To initially evaluate the anti-CRC activity of Pt517 in vitro, three colorectal cancer cell lines, CT26, HT29, Caco-2, and normal colonic epithelial cell line NCM460 were treated with the culture supernatant of Pt517 (Pt517CS) at 10–20% (*v*/*v*). As shown in Figure 1, Pt517CS treatment specifically and concentration-dependently inhibited the viability of CRC cells (*p* < 0.001). It was noteworthy that this inhibitory effect was particularly pronounced at concentrations of 14% or higher, and Pt517CS did not show significant toxicity to NCM460 cells within this concentration range (Figure 1A–D).

Colony formation assays showed that CT26 cell colonies in the Pt517CS group were significantly fewer than those in the control and *E. coli* MG1655 (EcCS) groups (*p* < 0.001; Figure 1E,F). Compared with the control and EcCS groups, Pt517CS reduced the wound healing efficiency of CT26 cells after 24 and 48 h (*p* < 0.001; Figure 1G,H).

### 3.2. Effect of Pt517CS on Apoptosis and Cell Cycle Arrest in CT26 Cells

**Figure 2:**
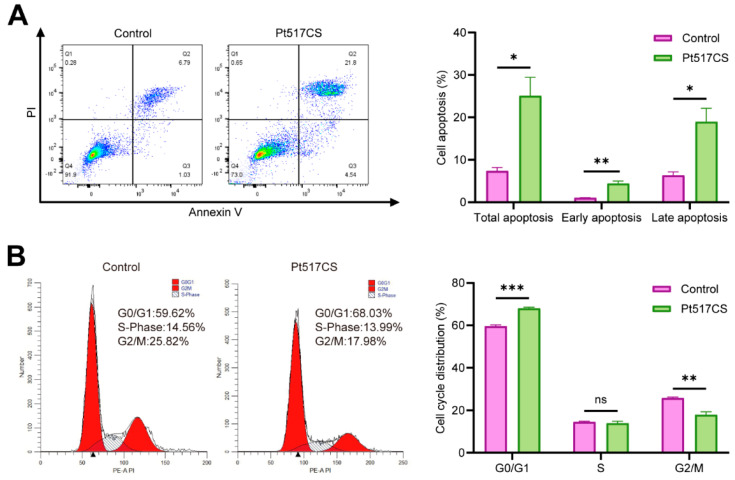
Pt517CS induces apoptosis and alters cell cycle progression in CT26 cells. (**A**) Treatment with Pt517CS for 48 h significantly promoted both early and late apoptosis in CT26 cells. (**B**) Following 48 h of Pt517CS treatment, CT26 cells underwent significant cell cycle arrest at the G0/G1 phase, concomitant with a marked decrease in the proportion of cells in the G2/M phase. Data are presented as means ± SEM. *n* = 3 biological replicates. *p* values are calculated by unpaired two-tailed Student’s *t*-test. ns, not significant, * *p* < 0.05, ** *p* < 0.01, *** *p* < 0.001.

To explore the mechanism by which Pt517CS inhibited CRC cell proliferation, we evaluated the effect of Pt517CS on apoptosis and cell cycle in CT26 cells. As shown in Figure 2A, Pt517CS treatment significantly increased the percentage of early and late apoptosis of CT26 cells, reaching 6.75% and 16.4%, respectively, compared with the control group (5.17% and 5.41%) (*p* < 0.05). Pt517CS treatment significantly increased the cell population in the G0/G1 phase and decreased those in G2/M phase, compared with the control group (*p* < 0.001, Figure 2B).

### 3.3. P. tenue Suppresses Tumor Growth in the CT26 Syngeneic Mouse Model

**Figure 3:**
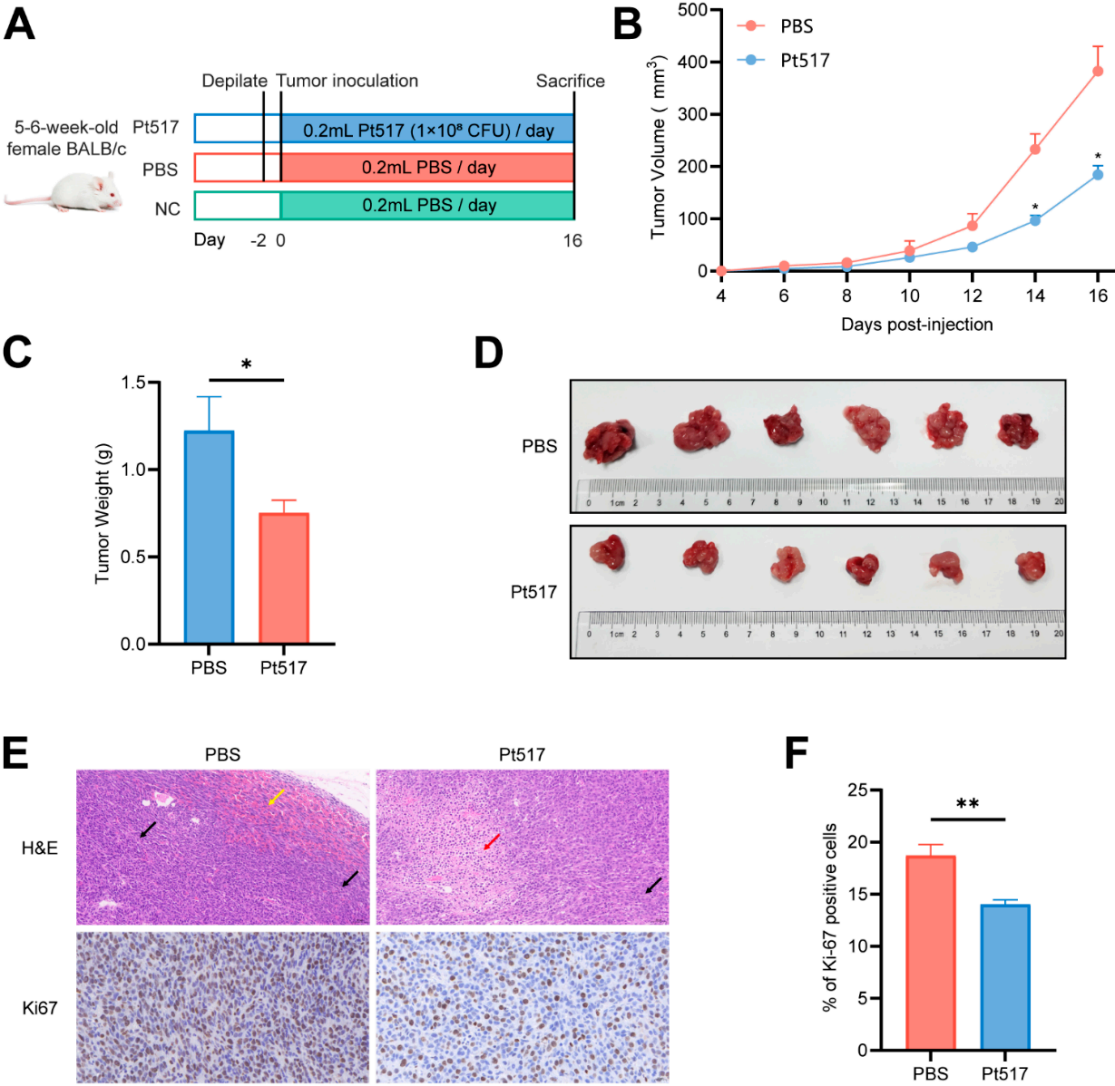
Antitumor efficacy of *P. tenue* in the CT26 syngeneic mouse model. (**A**) Experimental design for Pt517 treatment for CT26 syngeneic mouse model. (**B**) Daily oral administration of Pt517 significantly inhibited tumor growth. (**C**) Pt517 significantly reduced colorectal tumor weight in the CT26 syngeneic mouse model. (**D**) Representative images of tumors from CT26 syngeneic mouse model. (**E**) H&E staining for pathologic diagnosis (scale bar: 50 μm) and immunohistochemical (IHC) staining for Ki-67 (scale bar: 20 μm) of mouse tumors. The black arrows indicate the nuclear division, the red arrows indicate the necrosis area, and the yellow arrow indicates the inflammatory infiltration. (**F**) Quantitative analysis of Ki-67^+^ cells in the tumor tissues. Data are presented as means ± SEM, *n* = 6 for each group. Statistical differences were tested using two-way ANOVA with Bonferroni’s correction for post hoc testing in (**B**) and an unpaired two-tailed Student’s *t*-test in (**C**,**F**). * *p* < 0.05, ** *p* < 0.01.

The anti-CRC effect of PT517 was further validated in a CT26 syngeneic mouse model (Figure 3A). Administration of Pt517 significantly suppressed tumor growth by reducing tumor volume (*p* < 0.05) and weight compared to the PBS group (*p* < 0.05; Figure 3B–D).

Histopathological examination, as revealed by H&E staining, showed substantial differences between the groups. Tumor sections from the PBS group exhibited frequent mitotic figures and focal hemorrhages. In contrast, the Pt517-treated group exhibited extensive tumor cell necrosis, accompanied by a marked reduction in both mitotic figures and hemorrhage. Furthermore, the Pt517 group has a significantly lower number of Ki-67-positive cells, a marker of tumor proliferation, than in the PBS group (*p* < 0.01; Figure 3E,F).

### 3.4. P. tenue Alters the Expression of Immune Factors Within Tumors in the CT26 Syngeneic Mouse Model

**Figure 4:**
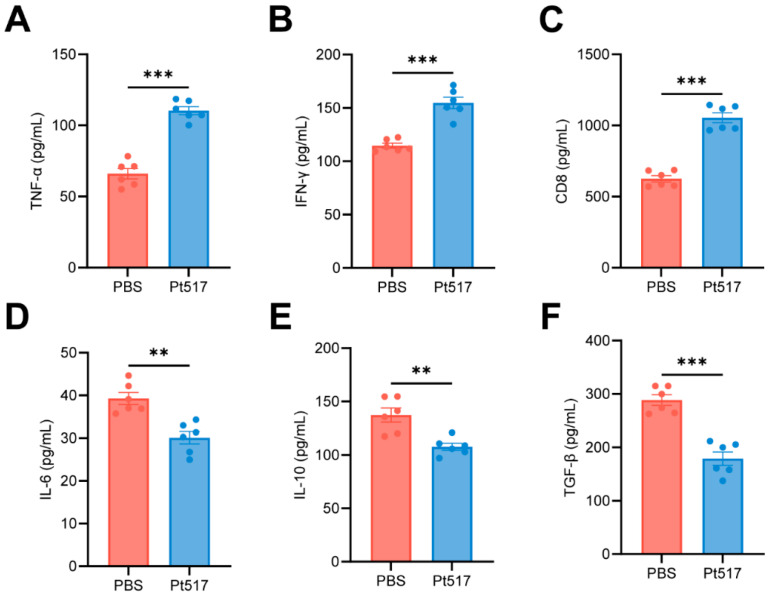
*P. tenue* modulates the expression of immune factors within tumors of CT26 syngeneic mouse model. Measurement of TNF-α (**A**), IFN-γ (**B**), CD8 (**C**), IL-6 (**D**), IL-10 (**E**), and TGF-β (**F**) in tumor tissue homogenates by ELISA. Data are presented as means ± SEM, *n* = 6 for each group. *p* values are calculated by unpaired two-tailed Student’s *t*-test. ** *p* < 0.01, *** *p* < 0.001.

To evaluate the immunomodulatory effects of Pt517, cytokine levels in tumor tissues were measured using ELISA. Compared with the PBS group, Pt517 treatment had significantly increased levels of antitumor cytokines, including TNF-α, IFN-γ, and the T-cell cytotoxic marker CD8 (*p* < 0.001), and significantly decreased levels of IL-6, IL-10, and TGF-β (*p* < 0.01, Figure 4A–F).

### 3.5. P. tenue Modulates the Gut Microbiota of the CT26 Syngeneic Mouse Model

**Figure 5:**
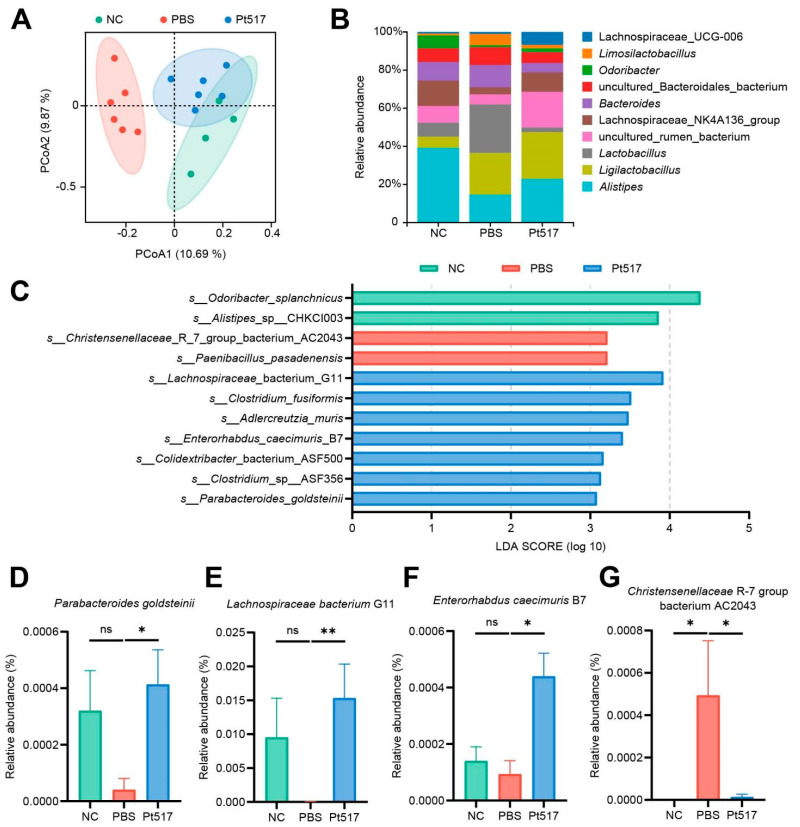
*P. tenue* modulates the gut microbiota of CT26 syngeneic mouse model. (**A**) PCoA based on the Binary-Chord dissimilarity matrix of OTU-level compositional profiles. Ellipses represent 95% CIs. (**B**) Bacterial community compositions at the genus level. (**C**) Distinct taxa were identified among groups by LEfSe analysis. Taxa meets a LDA score significant threshold > 3.0. (**D**–**G**) The relative abundances of *Parabacteroides goldsteinii* (**D**), *Lachnospiraceae bacterium* G11 (**E**), *Enterorhabdus caecimuris* B7 (**F**), and *Christensenellaceae* R7 group bacterium AC2043 (**G**) in different groups. Data are presented as means ± SEM, *n* = 4–6 biological replicates. *p* values are calculated by unpaired two-tailed Student’s *t*-test. ns, not significant, * *p* < 0.05, ** *p* < 0.01.

16S rRNA gene sequencing of the cecal contents from mice was performed to investigate the effect of Pt517 supplementation on gut microbiota. The 16 samples yielded a median of 12,504 clean reads. No significant differences in alpha diversity (as measured by the Chao and Shannon indices) were observed among the three groups (*p* > 0.05; Figure S1A,B). By PCoA of beta diversity, the compositional structure of the microbiota was markedly altered. The NC group was well separated from the Pt517 group, which overlapped partially with the PBS group (Figure 5A). Analysis of the taxonomic profiles at the genus level (Figure 5B) identified *Alistipes* as the most abundant genus in the NC group. In contrast, the PBS group was dominated by *Lactobacillus*, while the Pt517 group showed high abundance of *Ligurilactobacillus*.

LEfSe analysis (LDA score > 3.0) identified distinct microbial signatures for each group. The NC group was enriched with *Odoribacter splanchnicus* and *Alistipes* sp CHKCI003. The PBS group was characterized by *Christensenellaceae* R7 group AC2043 and *Paenibacillus pasadenensis*. In contrast, the Pt517 group exhibited a significant enrichment of multiple beneficial taxa, including *Lachnospiraceae* G11, *Clostridium fusiformis*, *Adlercreutzia muris*, *Enterorhabdus caecimuris* B7, *Colidextribacter* ASF500, *Clostridium* sp ASF356, and *Parabacteroides goldsteinii* (Figure 5C). Compared to the PBS group, the relative abundance of several Pt517-enriched bacteria, such as *P. goldsteinii*, *Lachnospiraceae* G11, and *E. caecimuris* B7, was significantly higher in both the NC and Pt517 groups. Conversely, the abundance of *Christensenellaceae* R7 group bacterium AC2043 was lower in the NC and Pt517 groups (*p* < 0.05; Figure 5D–G). Collectively, Pt517 affected the gut microbiota with enriched probiotic bacteria and reduced potential pathogens.

### 3.6. P. tenue Elevates Metabolites with Antitumor Effects in the Serum of the CT26 Syngeneic Mouse Model

**Figure 6:**
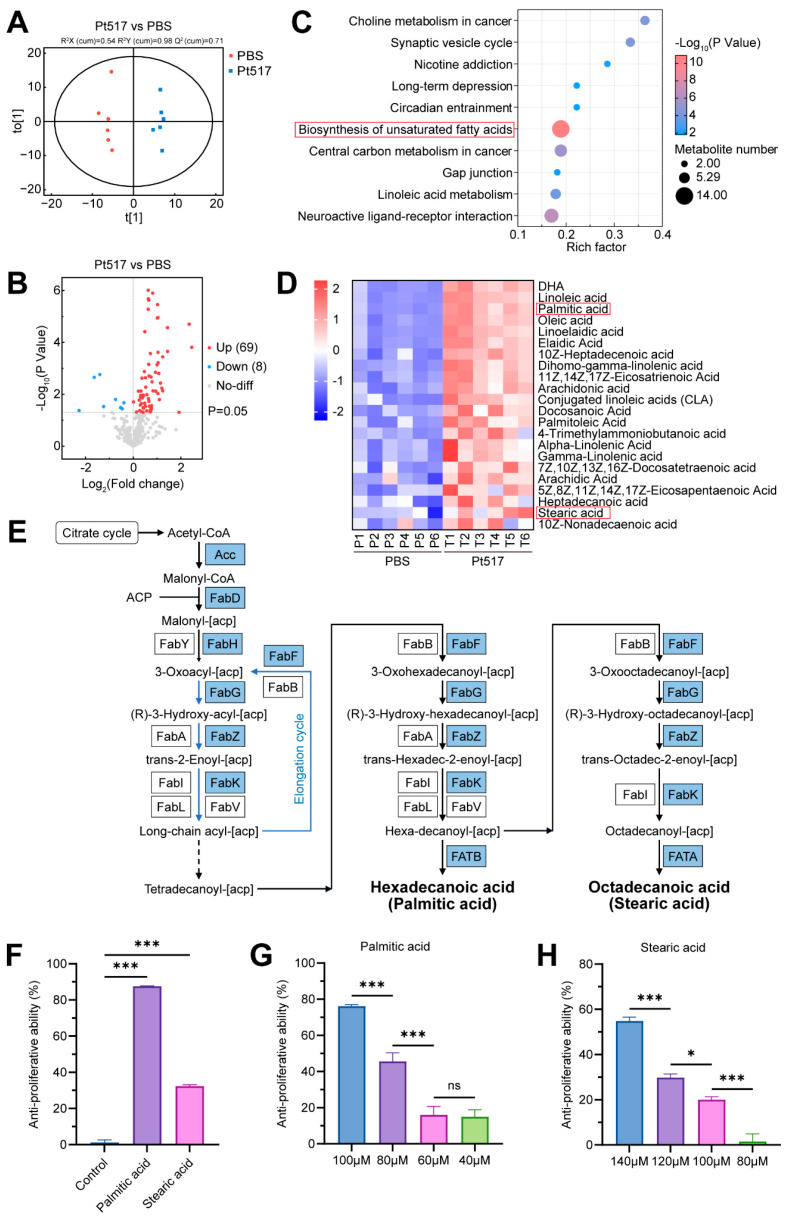
*P. tenue* alters the metabolites in the serum of CT26 syngeneic mouse model. (**A**) The differences in serum metabolic profiles between the PBS group and the Pt517 group by OPLS-DA analysis. (**B**) Volcano plots illustrated the detected metabolites in comparison with the PBS group and the Pt517 group. Red and blue dots represented significantly up-regulated metabolites (Fold change > 1, *p* < 0.05), and significantly down-regulated metabolites (Fold change < 1, *p* < 0.05) respectively. (**C**) KEGG functional enrichment analysis based on the differential metabolites in the PBS group and the Pt517 group. (**D**) Heatmap of the relative contents of the differential long-chain fatty acids identified in the PBS and Pt517 groups. (**E**) The evidence of key enzymes involved in the synthesis of palmitic acid and stearic acid by Pt517. (**F**) Palmitic acid and stearic acid suppressed the proliferation of CT26 cells. (**G**,**H**) Palmitic acid (**G**) and stearic acid (**H**) reduced the viability of CT26 cells in a concentration-dependent manner. Data are presented as means ± SEM, *n* = 6 for each group. *p* values are calculated by unpaired two-tailed Student’s *t*-test. ns, not significant, * *p* < 0.05, *** *p* < 0.001.

We performed serum metabolomics analysis on the sera of CT26 syngeneic mice to investigate metabolic alterations induced by tumor inoculation and the subsequent effects of Pt517 treatment. OPLS-DA models revealed distinct separations in the serum metabolite profiles among the different treatment groups (Pt517, PBS, and NC) (Figure 6A and Figure S2A), indicating significant metabolic changes. Volcano plots were used to visualize the differential metabolites in these comparisons (Figure S2B, Figure 6B). Tumor inoculation (PBS vs. NC) markedly disrupted the metabolome, resulting in the upregulation of 21 metabolites (including acylcarnitines, fatty acids, nucleosides, and phosphates) and the downregulation of 95 metabolites (primarily amino acids, peptides, and fatty acids) (Fold change > 1, *p* < 0.05). Crucially, Pt517 treatment largely reversed these changes. Compared to the PBS group, the Pt517 group showed upregulation of 69 metabolites (mostly amino acids, peptides, and fatty acids) and downregulation of only 8 metabolites (Fold change < 1, *p* < 0.05), suggesting a reversal of the tumor-induced metabolic profile toward a more normal state.

KEGG analysis was performed on differential metabolites between PBS and Pt517 groups. Among the top 10 significantly altered metabolic pathways identified between these two groups, the biosynthesis pathway of unsaturated fatty acids contained the highest number of differentially abundant metabolites (Figure 6C). All of the differential metabolites annotated in this pathway were long-chain fatty acids (LCFAs) (Table S1). A heatmap of the differential LCFAs between the PBS group and the Pt517 group was created to visualize the data (Figure 6D). The heatmap revealed 22 LCFA species, which were significantly increased in the serum of Pt517-treated mice. Levels of several LCFA were significantly higher in the Pt517 group than in the PBS group, including palmitic acid, stearic acid, conjugated linoleic acid (CLA), γ-linolenic acid, and heptadecanoic acid (Figure 6C). Among these LCFAs, palmitic acid and stearic acid were also detected in the Pt517CS (Table S2). In addition, the genome of Pt517 was found to encode the genes for the synthesis of LCFAs (such as palmitic acid and stearic acid) from acetyl-CoA (Figure 6E, Table S3). Specifically, its genome encodes key rate-limiting enzymes involved in the synthesis of palmitic acid and stearic acid, including acetyl-CoA carboxylase (Acc), malonyl-CoA: ACP transacylase (FabD), 3-ketoacyl-ACP synthetase III (FabH), enoyl-ACP reductase (FabK), and β-ketoacyl-ACP synthase II (FabF). Thus, functional prediction of the genes encoding these key enzymes indicates that Pt517 could synthesize palmitic acid and stearic acid.

Subsequently, we investigated whether palmitic acid and stearic acid inhibit the proliferation of CT26 cells in vitro. The results showed that, compared with ethanol solvent (control), both palmitic acid and stearic acid significantly reduced the viability of CT26 cells (*p* < 0.001; Figure 6F), and this inhibitory ability was dose-dependent (Figure 6G,H). These results demonstrated that the anti-CRC effect of Pt517 may be mediated through palmitic acid or stearic acid.

## 4. Discussion

In this study, we examined the antitumor effect of *P. tenue* strain Pt517 by testing the effect of *P. tenue* on CRC cells in vitro and in the CT26 syngeneic mouse model. We found that *P. tenue* Pt517CS specifically and dose-dependently inhibited the growth of CRC cells, promoted the apoptosis of CT26 cells, and induced the cell cycle arrest at the G0/G1 phase. Our findings are consistent with the established anticancer properties of microbial supernatants from other species [41,42,43]. For example, the exopolysaccharides from *Kluyveromyces marxianus* and *Pichia kudriavzevii* selectively reduced the viability of different CRC cell lines and induced the apoptosis of CRC cells by obstructing the AKT-1, mTOR, and JAK-1 pathways [41]. Similarly, *P. kudriavzevii* AS-12 metabolites [42] and the culture supernatant of *Lactobacillus reuteri* [43] can inhibit proliferation and trigger intrinsic apoptosis pathways. While our results are consistent with this broad mechanism of action, the precise molecular pathways by which Pt517CS induces apoptosis warrant further elucidation. Our study revealed that the gavage of *P. tenue* Pt517 bacteria significantly inhibited the growth of colorectal tumors in CT26 syngeneic mice. This finding is in alignment with other reports that probiotics could inhibit tumor growth in the CT26 syngeneic mouse model [44,45].

The inhibition of the CRC tumor growth by Pt517 was likely due to LCFAs produced by Pt517 or associated with Pt517 treatment. Genomic analysis revealed that Pt517 encodes essential enzymes for the synthesis of LCFAs, including stearic acid and palmitic acid. Pt517 produced palmitic acid and stearic acid, as they were detected in the Pt517CS. Other probiotic strains have been reported to have a similar effect through LCFAs. *L. plantarum* CCFM8661 ameliorated CRC by producing conjugated linoleic acid (CLA), which inhibited the NF-κB pathway and pro-inflammatory cytokines, upregulated tight junction proteins (ZO-1, claudin-1) and MUC2, and promoted tumor cell apoptosis in a PPAR-γ-dependent manner [46]. *L. plantarum* inhibited CRC in vivo and in vitro by producing γ-linolenic acid to induce mitochondrial damage and trigger ferroptosis [47].

In our study, treatment of Pt517 significantly increased serum levels of the LCFAs (stearic acid, palmitic acid, CLAs, DHA, and γ-linolenic acid) in mice. Specifically, both stearic acid and palmitic acid were found to directly inhibit the proliferation of CRC cells. Similarly, it has been reported that stearic acid significantly inhibits the proliferation of human colorectal cancer cell lines HCT116 and Caco2 in a dose-dependent manner, thereby suppressing CRC tumor growth in an AOM/DSS CRC mouse model [48]. Palmitic acid also induces apoptosis in HCT116 cells by activating the p53 signaling pathway and upregulating its downstream targets, p21 and Sesn2, in both dose- and time-dependent manners [49]. Additionally, palmitic acid acts as a potential antitumor molecule and exerts inhibitory effects against multiple cancers such as gastric, hepatic, cervical, breast, and colorectal cancers [50,51]. CLAs have been widely reported to exhibit anti-CRC effects via multiple mechanisms, such as regulating inflammation, cell proliferation, and apoptosis [52,53,54]. Therefore, Pt517 may inhibit CRC by secreting LCFAs, especially palmitic acid and stearic acid.

The detection of CLA, γ-linolenic, and heptadecanoic acid in the serum of Pt517-treated mice suggests that there were likely other sources of LCFAs, as these were not detected in the supernatant of Pt517. Previous studies have confirmed that specific gut microbiota can synthesize and metabolize such LCFAs [55,56,57]. Thus, Pt517 may regulate microbiota composition, induce the metabolism of intestinal flora to produce these LCFAs through interaction with the gut microbiota, and ultimately contribute to the regulation of its anti-CRC effect. Moreover, several studies confirm the critical role of the gut microbiota in LCFA synthesis. *Bifidobacteria* and *Propionibacteria* strains can produce conjugated α-linolenic, γ-linolenic, and stearic acid [55]. Several intestinal bacteria (such as *Enterococcus, Lactobacillus, Ruminococcus*, and *Lachnospiraceae*) can convert dietary linoleic acid into CLAs through linoleic acid isomerases [57]. These studies further support the hypothesis that elevation of some LCFAs in Pt517-treated mice was mediated by the gut microbiota.

Studies have shown that probiotics can alter the composition of gut microbiota to arrest cancer progression [14,27,45,58]. For instance, *Lacticaseibacillus rhamnosus* and *L. gallinarum* augmented the antitumor response to anti-PD-1 therapy in CT26 syngeneic mice by increasing commensal beneficial bacteria, including *Lactobacillus helveticus*, *Lactobacillus reuteri, L. rhamnosus*, *Lactobacillus Johnsoii*, and *Bifidobacterium animalis* in the gut microbiome [27,44,45]. *L. lactis* HkyuLL 10 and *Streptococcus thermophilus* suppressed tumourigenesis in CRC mice, also by enriching probiotic bacteria in the gut microbiota [14,58]. Similarly, in this study, we found that Pt517 altered the intestinal microflora structure of the CT26 syngeneic mice. Pt517 intervention significantly enriched the abundance of *P. goldsteinii*, *Lachnospiraceae* bacteria, and *E. caecimuris* B7, and depleted *Christensenellaceae* R7 group bacterium AC2043. It has been reported that *P. goldsteinii* can inhibit CRC cell growth in vitro [59], restrict the CRC liver metastasis in vivo [60], and secrete anticancer SCFAs [61,62]. The *Lachnospiraceae* family showed a relatively high abundance in normal colon tissues, while their abundance was significantly decreased in CRC tumor tissues [63,64]. Bacteria in this family degrade lysoglycerophospholipids in the tissue microenvironment to activate CD8^+^ T cell immune surveillance, thereby suppressing colon cancer progression [65]. Moreover, *Lachnospiraceae* can also produce palmitic acid and stearic acid with anti-CRC effects [48,56]. *E. caecimuris* was significantly reduced in the early and late stages of CRC and was enriched in the normal human intestine [66]. Whereas *Christensenellaceae* R-7 group bacterium was enriched in the intestine of patients with CRC, and it was considered a microbial marker associated with CRC adenoma [67]. These studies demonstrated that Pt517 increased the abundance of bacterial species with anti-CRC effects while decreasing the abundance of species associated with a heightened risk of CRC.

Numerous studies have demonstrated that probiotics can suppress tumorigenesis by regulating antitumor immune response [27,51,68,69,70,71,72]. *Bifidobacterium* inhibited the growth of colorectal tumors by increasing the proportion of CD8^+^ T cells in tumor tissues, especially the proportions of INF-γ^+^ CD8^+^ T cells and TNF-α^+^ CD8^+^ T cells [68]. In the CT26 syngeneic mouse model, *L. rhamnosus* Probio-M9 inhibited tumor growth by increasing the expression of antitumor cytokine IFN-γ and reducing the expression of immunosuppressive cytokine IL-10 in tumor tissues [27]. Certain *Bifidobacterium* spp. significantly reduced the level of the immunosuppressive cytokine IL-6 in tumor tissues [73]. Similarly, our study found that Pt517 significantly increased the levels of antitumor cytokines (TNF-α, INF-γ, and CD8) and significantly decreased the levels of immunosuppressive cytokines (IL-6, IL-10, and TGF-β). Additionally, studies have shown that certain LCFAs can exert anti-tumor effects by modulating immune responses. For instance, palmitic acid could exert anti-CRC effects by blocking the polarization of M2-polarized tumor-associated macrophages and the epithelial–mesenchymal transition of CRC cells, increasing the expression of TNF-α, and decreasing the expression of IL-10 in CRC cells [51,70,72]. Stearic acid could significantly increase the secretion of TNF-α in human THP-1 monocytes [74]. Consequently, we hypothesize that Pt517 reverses the tumor immunosuppressive state by producing LCFAs, particularly stearic acid and palmitic acid, thereby suppressing CRC tumor growth.

Despite the promising findings, several limitations of the study should be acknowledged. First, the antitumor effect of Pt517 has so far only been confirmed in the CT26 syngeneic mouse model, and further validation is needed in Apc^Min/+^ C57BL/6 mice, a spontaneous colorectal cancer model that accurately recapitulates human familial adenomatous polyposis. Second, a germ-free mouse model is required to determine whether gut microbiota are necessary for Pt517 to exert its antitumor activity. Finally, to establish a definitive causal relationship, a deficiency of key enzymes involved in LCFA biosynthesis is required to verify the precise role of LCFAs in mediating the observed anti-tumor effects.

In conclusion, this study revealed that *P. tenue* Pt517 possesses anti-CRC activity. In vitro, Pt517CS directly inhibited the proliferation and clone formation of CRC cells, while inducing cell apoptosis and altering the cell cycle. In the CT26 syngeneic mouse model, Pt517 inhibited tumor growth through suppressing the expression of the tumor proliferation marker Ki-67, activating the anti-tumor immune response, reshaping the structure of the intestinal flora, and modulating the metabolites in the mice serum, especially LCFAs with anticancer effects, such as stearic acid and palmitic acid. These findings not only elucidated the anti-tumor mechanism of *P. tenue* but also provided a new candidate for the microbial treatment of CRC.

## Data Availability

The original contributions presented in this study are included in the article/Supplementary Material. Further inquiries can be directed to the corresponding authors.

## Supplementary Materials

The following supporting information can be downloaded at: https://www.mdpi.com/article/10.3390/cells15090805/s1, Figure S1: Alpha diversity of gut microbiota in 3 groups of mice; Figure S2: Analysis results of serum metabolites in CT26 syngeneic mouse model; Table S1: The metabolites enriched in the KEGG pathway of biosynthesis of unsaturated fatty acids; Table S2: The content of differential metabolites between Pt517CS and RCM; Table S3: The key enzyme evidence for the synthesis of palmitic acid and stearic acid by Pt517.

## Abbreviations

The following abbreviations are used in this manuscript:

CFU	colony-forming units
CLA	conjugated linoleic acid
CRC	colorectal cancer
EcCS	the culture supernatant of *E. coli* MG1655
ELISA	enzyme-linked immunosorbent assay
H&E	hematoxylin-eosin
LCFAs	long-chain fatty acids
LEfSe	linear discriminant analysis effect size
NCBI	National Center for Biotechnology Information
OPLS-DA	orthogonal partial least-squares discriminant analysis
Pt517CS	the culture supernatant of *P. tenue* Pt517
RCM	reinforced clostridial medium
SCFAs	short-chain fatty acids
VIP	variable importance in the projection
