# Supplementary material for: Paraclostridium tenue Exhibits Antitumor Activity Through Generating Antitumor Metabolites and Modulating Gut Microbiota
Source: Cells. 2026 Apr 29;15(9):805. doi: 10.3390/cells15090805 (PMC13162938; doi:10.3390/cells15090805)
Supplement: Supplementary file 1 [file cells-15-00805-s001.zip › Supplementary Material.pdf]

Supplementary Material

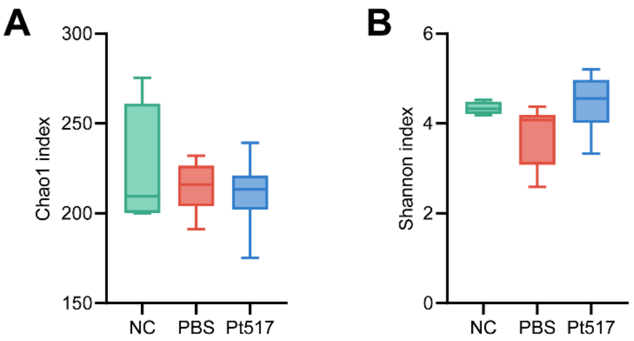

Figure S1. Alpha diversity of gut microbiota in 3 groups of mice. Chao 1 index (A) and Shannon index (B) of gut microbiota in each group of mice. Data are presented as means ± SEM, n=4-6. P values are calculated by One-way ANOVA with Tukey's correction for post hoc testing.

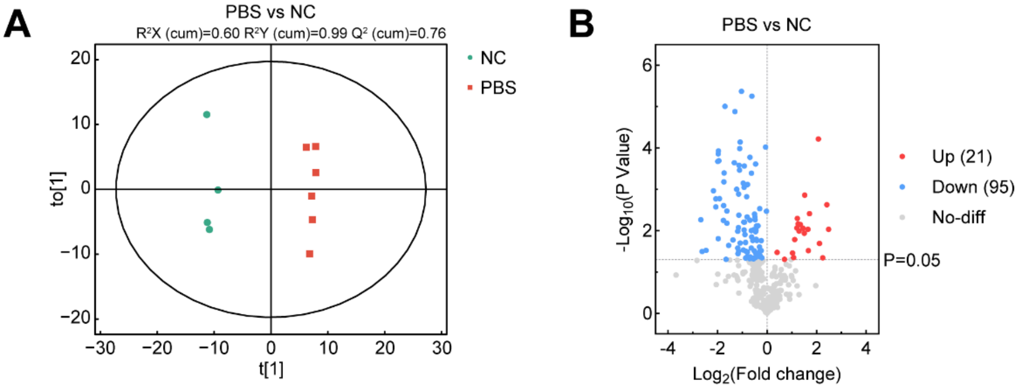

Figure S2. Analysis results of serum metabolites in CT26 syngeneic mouse model. (A) Serum metabolic profile significantly differed between the NC group and the PBS group mice by OPLS-DA analysis. (B) Volcano plots illustrated the detected metabolites in comparison with the NC group and the PBS group.
